# Characterization of Two ENU-Induced Mutations Affecting Mouse Skeletal Morphology

**DOI:** 10.1534/g3.113.007310

**Published:** 2013-10-01

**Authors:** Shauna M. Dauphinee, Megan M. Eva, Kyoko E. Yuki, Melissa Herman, Silvia M. Vidal, Danielle Malo

**Affiliations:** * Department of Human Genetics, McGill University, Montreal, Quebec H3G 0B1, Canada; † Complex Traits Group, McGill University, Montreal, Quebec H3G 0B1, Canada; ‡ Department of Medicine, McGill University, Montreal, Quebec H3G 0B1, Canada

## Abstract

Using the *N*-ethyl-*N*-nitrosourea (ENU) mutagenesis screen, we have identified two skeletal morphology mutants, *Skm1* and *Skm2*. Positional cloning and candidate gene sequencing localized the causative point mutations within the genes coding for natriuretic peptide receptor C (NPR-C) and filamin b (FLNB), respectively. Mice that carry a mutation in *Npr3* exhibit a skeletal overgrowth phenotype, resulting in an elongated body and kyphosis. *Skm2* mice, carrying a mutation in *Flnb*, present with scoliosis and lordosis. These mutant mice will serve as useful models for the study of vertebral malformations.

Vertebral malformations that cause kyphosis or scoliosis are an important health problem that can result in disability and suffering attributable to both physical pain and psychological distress from the disfigurement. Congenital spine deformities occur with an approximate frequency of 0.5–1 per 1000 live births (Sparrow *et al.* 2011) and may arise sporadically or as the result of an underlying heritable abnormality in genes that contribute to vertebral development. Although there is evidence that the embryonic environment, including oxygen tension, exposure to teratogens, and prenatal vitamin deficiencies, can all contribute to congenital defects (Hensinger 2009), the genetic contribution to the pathogenesis of vertebral malformation syndromes is less understood. This is largely attributable to the fact that, in humans, vertebral malformation syndromes often represent a sporadic occurrence within a family and traditional linkage analysis to identify the chromosomal regions responsible for these defects is often not possible. As a result, the genetic etiology of many congenital vertebral malformation syndromes remains unknown. However, familial occurrences (Miller *et al.* 2006; Wynne-Davies 1975) and twin studies (Kesling and Reinker 1997) have shown that the cause of spinal deformities, including scoliosis and kyphosis, can have a genetic component.

Using model organisms and using a phenotype-driven approach to identify causal genes underlying spinal deformities can overcome some of the limitations to studying the genetic basis of these syndromes. Mouse models have contributed greatly to our understanding of molecular embryonic development, including vertebral formation and segmentation (Sparrow *et al.* 2011), and the use of forward genetics screens has been shown to be a valuable tool for the discovery of mutations that are important during skeletogenesis in the mouse (Mohan *et al.* 2008). *N*-ethyl-*N*-nitrosourea (ENU) is a chemical mutagen that introduces random point mutations into the mouse genome that, together with positional cloning or exome sequencing, permits the identification of phenotype–genotype relationships in an unbiased manner (Probst and Justice 2010).

In this article, we report the identification of two mutations affecting skeletal morphology, *skeletal morphology 1* (*skm1*) and *skeletal morphology 2* (*Skm2*), in a recessive ENU mutagenesis screen for infectious disease susceptibility. Homozygous *Skm1* mice show kyphosis or an elongated torso and homozygous *Skm2* mice exhibit shortened stature, scoliosis, and lordosis. We show that *Skm1* and *Skm2* mice carry a missense mutation in the *Npr3* and *Flnb* genes, respectively. These mutants provide important tools for the study of skeletal malformation disorders that result from the loss of a functional *Npr3* or *Flnb* gene.

## Materials and Methods

### Mice

The 129S1/SvImJ (129S1) males, 129X1/SvJ (129X1) females, and DBA/2J mice were purchased from the Jackson Laboratory (Bar Harbor, ME) at age 6 wk. Mice were housed at the McGill University Life Sciences Centre Animal Facility in accordance with the guidelines specified by the Canadian Council on Animal Care. The Animal Use Protocol was approved by the McGill University Animal Care Committee.

### ENU mutagenesis

Male 129S1 mice received one intraperitoneal injection of ENU at a dose of 150 mg/kg at age 8–12 wk. Infertility of the mutagenized mice was confirmed after treatment and mice were allowed 8–12 wk to recover fertility and then were bred to 129X1 females. Mice were outcrossed to DBA/2J at the first generation (G1) to facilitate mapping using a panel of markers polymorphic between 129S1 and DBA/2J strains, and second generation (G2) progeny were backcrossed to G1 mice to generate homozygosity. Mice with visible abnormalities in the third generation (G3) were kept for further analysis.

### Genotyping/mapping

DNA was isolated from tail biopsy specimens using overnight digestion in a lysis buffer containing proteinase K (10  nM Tris, 2  mM EDTA, 0.5% SDS, 0.4  M NaCl, and 0.2  mg/ml proteinase K), followed by a phenol/chloroform extraction. The genome scan was conducted using an Illumina Mouse Medium density SNP panel (Genetic Analysis Facility, Centre for Applied Genomics, Hospital for Sick Children, Toronto, ON). Chromosome distances were established using NCBI build 37.1.

### Genetic analysis

J/QTL software (http://churchill.jax.org/software/jqtl.shtml) was used to calculate LOD scores for the whole-genome SNP analysis. The software assembled a genetic map from the SNP data using the settings of binary model, expectation-maximum algorithm, and 1000 permutations.

### Sequence analysis

Exons of candidate genes were sequenced for wild-type and mutant mice, as well as parental controls, at the McGill University and Génome Québec Innovation Centre.

### X-ray measurement

Mice (age approximately 7 wk) were anesthetized using a combination of 100 mg/kg ketamine HCl, 10 mg/kg xyaline HCl, and 3 mg/kg acepromazine administered by subcutaneous injection and radiographs were performed using a Faxitron (Hewlett Packard, Palo Alto, CA).

## Results

### Identification of the *Skm* mutants

**Figure 1:**
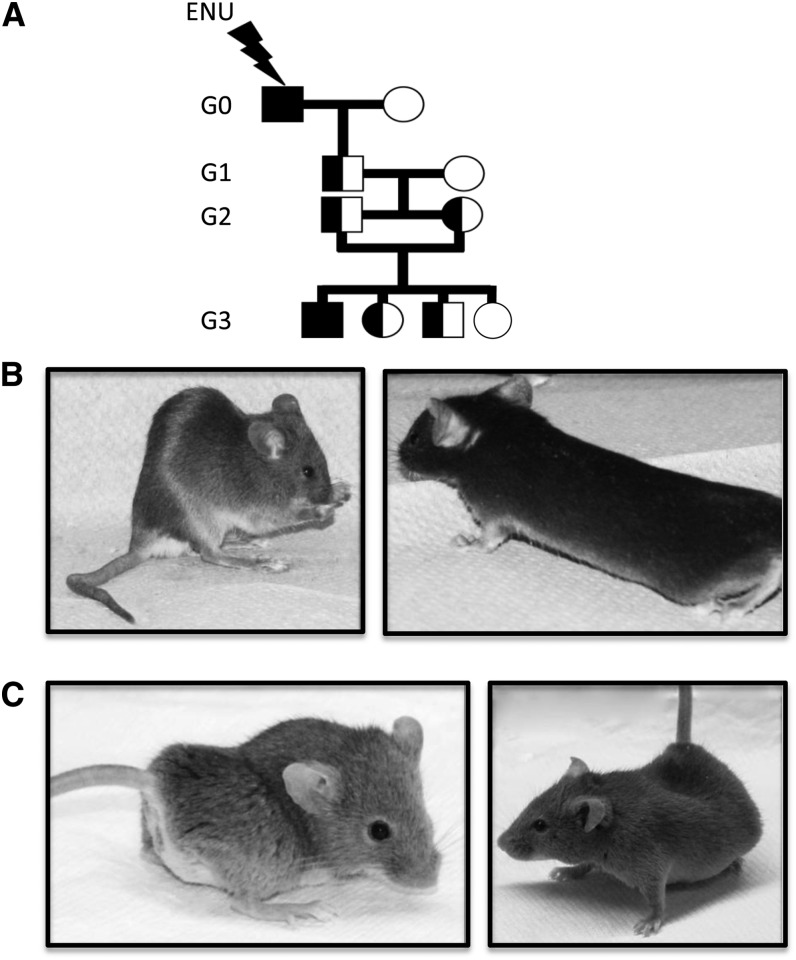
*N*-ethyl-*N*-nitrosourea (ENU) breeding scheme and visible morphology of skeletal morphology mutants (*Skm*). (A) Schematic representation of the breeding scheme used during the identification of deviant pedigrees. 129S1 male G0 mutagenized mice were crossed to 129X1 females and the resulting G1 males were outcrossed to DBA/2J females to generate G2 progeny. Visible skeletal malformations in *Skm1* (B) mice and *Skm2* (C) mice. (B) *Skm1* mice show kyphotic spines and kinked tails (*left*) and elongated bodies (*right*). (C) *Skm2* mice exhibit severe scoliosis and lordosis.

During our ENU mutagenesis screen for infectious disease susceptibility, we identified two mutations that contribute to skeletal malformations in mice. In our pipeline, ENU-mutagenized mice were bred to homozygosity in a three-generation breeding scheme (Figure 1A), and although G3 progeny were to be screened for susceptibility to bacterial infection, mice were initially examined for gross morphological defects. Here, we report the identification of two pedigrees based on their skeletal disfigurement, *Skm1* and *Skm2*.

*Skm1* homozygous mutant mice were born with expected Mendelian frequency and were easily distinguished from their wild-type littermates at weaning. *Skm1* mice were visibly leaner than their littermate controls and presented with either an elongated body or a thoracolumbar kyphosis and a kinked tail (Figure 1B). *Skm2* mice presented a more severe phenotype and were readily identified after birth by a shortened stature, abnormal gait, and severe scoliosis and lordosis (Figure 1C). In addition, *Skm2* mutants were dehydrated and presented with tachypnea, likely as a consequence of thoracic deformities. At weaning, only eight *Skm2* mutants out of 84 progeny were identified (9.5% ratio), suggesting embryonic or perinatal mortality.

### Positional cloning of the *Skm* mutants

**Figure 2:**
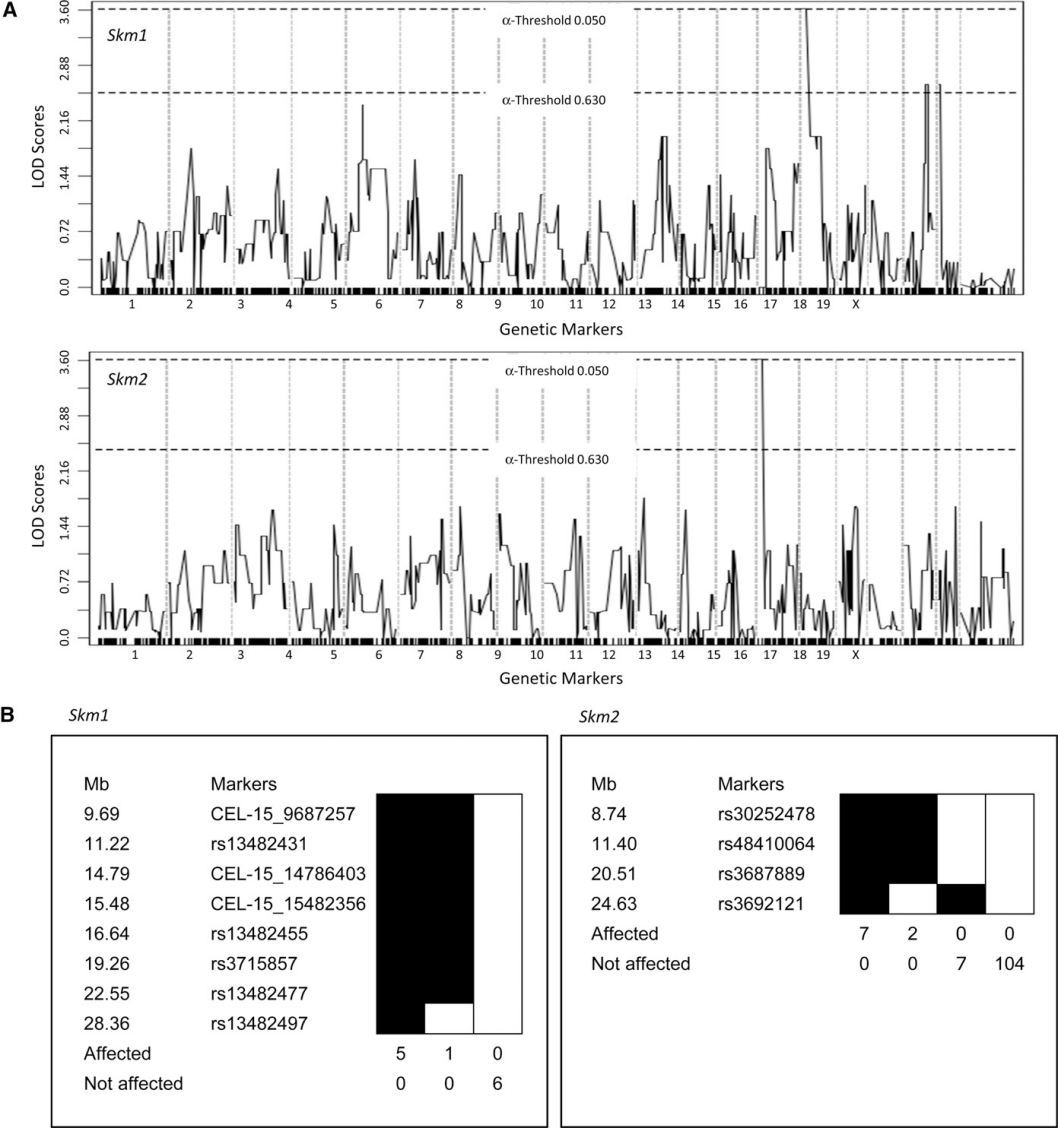
Mapping of the mutant pedigrees. (A) Genome-wide linkage analysis of the visible mutants (*Skm1*, *left panel*; *Skm2*, *right panel*) was conducted with 12 animals (6 mutant and 6 normal) using polymorphic markers informative for the 129S1 and DBA/2J parents. LOD scores above the threshold line were considered significant. (B) Haplotype analysis of the proximal region of chromosome 15 for *Skm1* (*left panel*) and of chromosome 14 for *Skm2* (*right panel*). Positions of the markers (Mb) are relative to the centromere.

**Figure 3:**
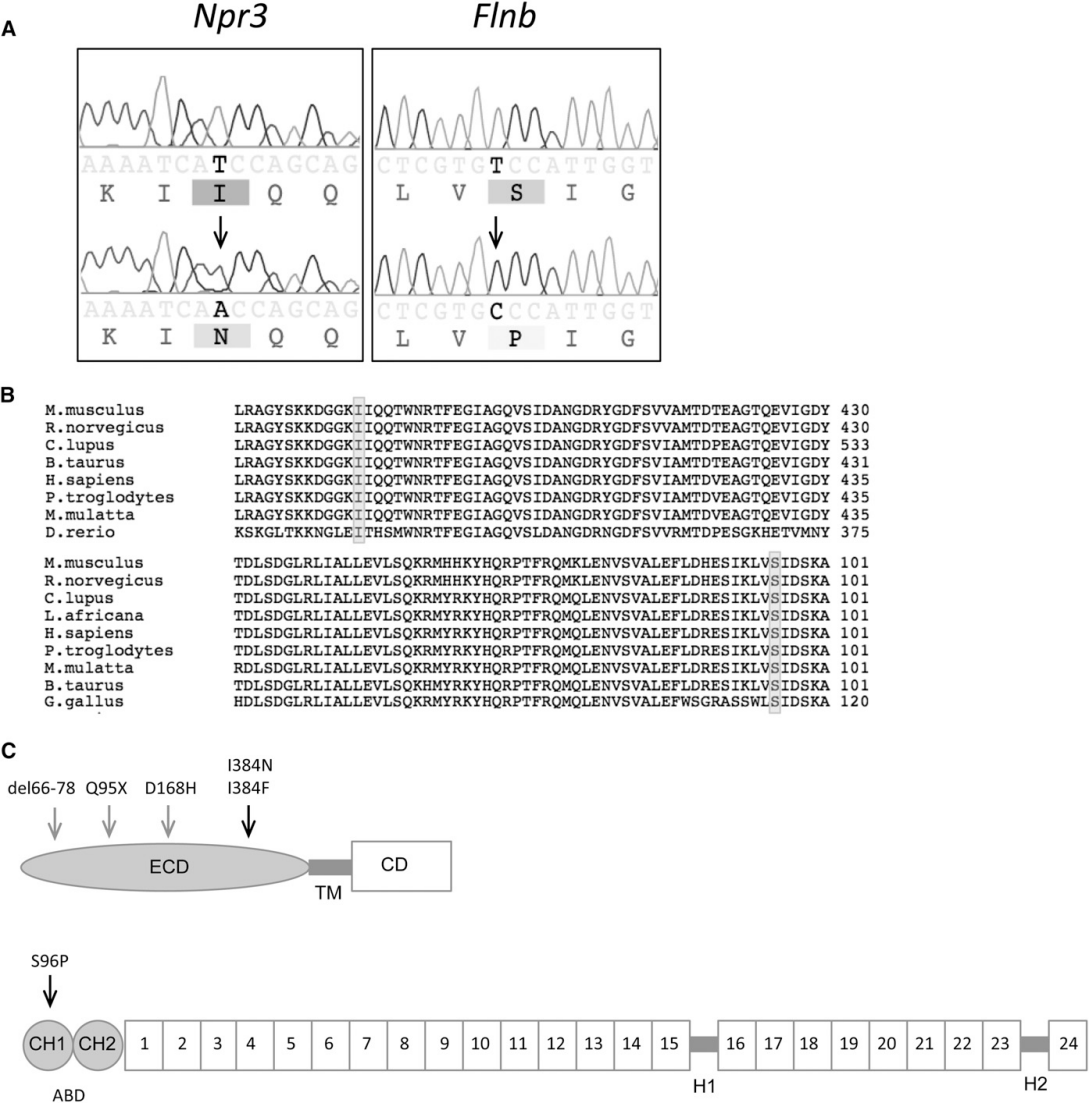
Identification of mutations underlying skeletal morphology mutant 1 (*Skm1*) and skeletal morphology mutant 2 (*Skm2*). (A) Genomic DNA sequence chromatograms from normal (*upper*) and mutant (*lower*) *Skm1* and *Skm2* mice (*left panel* and *right panel*, respectively). The location of the mutation is indicated by the arrowhead. (B) Alignment of the amino acid sequence for NPR3 (*top*) and FLNB (*bottom*) orthologs. The location of the mutation is indicated by shading. (C) Schematic representation of NPR3 (*top*) and FLNB (*bottom*).The arrows indicate location of *Skm* mutations (black) and other reported mutations (gray). NPR3 contains an extracellular domain (ECD), transmembrane (TM) domain, and cytoplasmic domain (CD). FLNB comprises an actin-binding domain (ABD) containing two calponin homology (CH) domains at the amino terminus, followed by 24 filamin repeat domains that are separated by two hinge (^1^H-2) regions.

The *Skm* mutations originally generated on a 129S1 background were segregated on a mixed genetic background of 129S1, 129X1, and DBA/2J. For mapping, a total of six mice for each of the visible phenotypes were used in a SNP panel with 708 markers polymorphic between 129 and DBA/2J strains across the entire genome. The *Skm1* mutants generated a significant linkage (LOD score = 3.612) at chromosome 15 at marker rs13482455 (16.73 Mb) (Figure 2A, *top panel*). The region of the mutation was localized to a 22.6 Mb interval on chromosome 15 (Figure 2B, *left panel*) containing 180 known genes. Candidate genes were prioritized based on reported phenotypes and natriuretic peptide receptor C (*Npr3*) was selected for sequencing based on the existence of seven known alleles (spontaneous and generated) with phenotypes similar to those of the *Skm1* mice. Sequencing of genomic DNA revealed a T-to-A transversion at nucleotide position 1151, causing a mutation from an isoleucine residue to an asparagine residue (I384N) (Figure 3A, *left panel*). The mutation underlying the *Skm1* phenotype occurred at a residue that is highly conserved across species (Figure 3B, *upper panel*) and mouse strains (data not shown). This mutation occurs within the membrane proximal region of the extracellular domain of the receptor, which is important for ligand binding (Figure 3C, *upper panel*).

The *Skm2* mutants generated a significant linkage (LOD score = 3.612) at chromosome 14 at marker rs3687889 (19.69 Mb) (Figure 2A, *bottom panel*). Genotyping of 120 mice, including 10 mice with a deviant phenotype, confined the mutation to a region of 24.6 Mb on chromosome 14 (Figure 2B, *right panel*), containing 394 known/predicted genes and 3 genes with the associated mouse phenotype of kyphosis [*Ataxin 7*, *Topoisomerase* (*DNA*) *II beta*, and *Filamin b*]. DNA sequence analysis of *Flnb* revealed a T-to-C transition at nucleotide position 481 converting a serine residue to a proline residue (S96P), resulting in a missense mutation (Figure 3A, *right panel*) within the actin-binding domain of FLNB (Figure 3C, *bottom panel*). All mice that carry the mutation display the skeletal phenotype (data not shown). In this case also, the mutation occurred at a residue highly conserved across species (Figure 3B, *bottom panel*) and mouse strains (data not shown).

### Examination of the skeletal abnormalities

**Figure 4:**
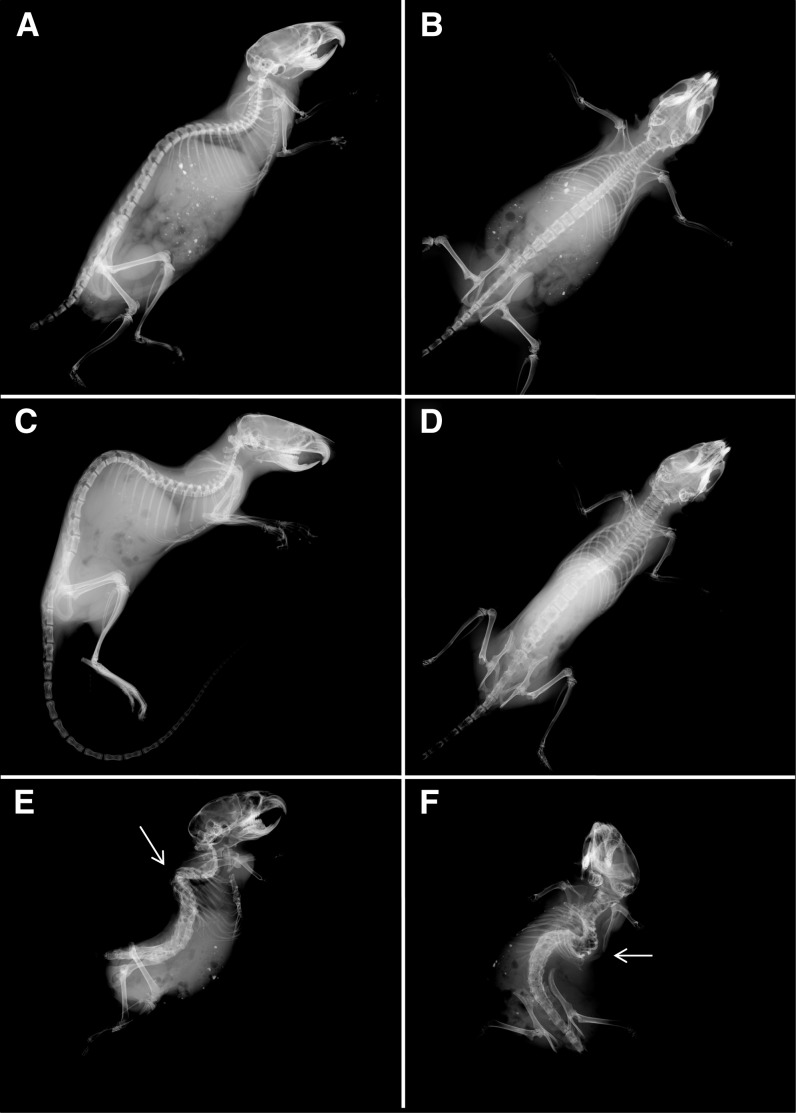
Skeletal abnormalities in skeletal morphology mutant (*Skm)* mice. Radiographs of normal (A, B), *Skm1* (C, D), *and Skm2* (E, F) mice in lateral and supine positions. *Skm1* mice in the lateral position showing kyphosis (C) and in the supine position showing elongated body (D). *Skm2* mice in the supine (E) and lateral (F) positions. Scoliotic and lordotic curvatures of the vertebral columns are indicated by arrowhead relative to wild-type littermates.

**Figure 5:**
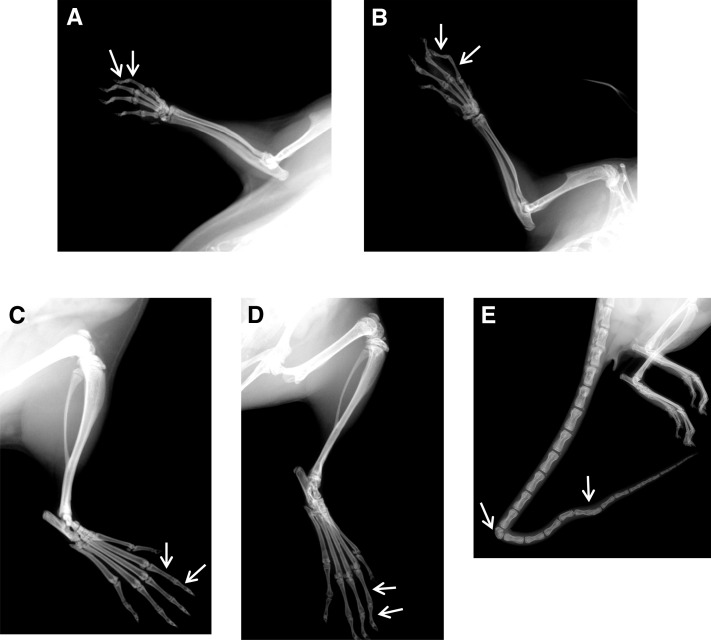
Arachnodactyly and kinked tail in skeletal morphology mutant 1 (*Skm1*). Radiographs of normal (A–C) and *Skm1* (B–D) manus (A, B) and pes (C, D). Phalanges are indicated by arrowheads. (E) Radiograph of the tail of *Skm1*. The hemivertebrae and fused vertebrae are indicated by arrowheads.

To further characterize the skeletal abnormalities, we performed radiography on wild-type and mutant mice aged 6 wk. *Skm1* mice were thin and exhibited a marked thoracolumbar kyphosis (Figure 4, C and D) compared to wild-type littermates (Figure 4, A and B). *Skm1* mice also exhibited arachnodactyly characterized by elongated phalanges (Figure 5, B and D), compared to wild-type littermates (Figure 5, A and C), and kinked tail (Figure 5B), attributable to either the presence of hemivertebrae or fused vertebrae in the tail region (Figure 5E). *Skm2* mice exhibited abnormal skeleton morphology, with severe scoliosis (the convexity of the curve pointing to the left) and lordosis (Figure 4, E and F). In addition, the mutant mice showed an abnormal thoracic cage with less ribs, a malformed sternum, and fusion of several vertebrae.

## Discussion

Using a large-scale ENU mutagenesis screen, we have identified two novel mutations, *Skm1* and *Skm2*, that result in congenital spinal deformities in mice. The genes underlying these new ENU-derived mutants have previously been associated with skeletal abnormalities in both mice and humans, and thus serve as excellent tools for the study of the human skeleton and its development.

The *Skm1* mutation lies within the *Npr3* gene coding for natriuretic peptide receptor C (NPR-C). Natriuretic peptides (NP), which are important in cardiovascular homeostasis through the maintenance of blood pressure and extracellular fluid volume, comprise three structurally related molecules: atrial NP, brain NP, and C-type NP (Potter 2011). Osteocrin, a bone-secreted protein important in osteoblast regulation, shows homology to the NP family and can also bind to NPR-C (Moffatt *et al.* 2007). NPR-C acts as a clearance receptor for these peptides through an endocytic mechanism that involves recycling of the receptor at the cellular membrane (Rubattu *et al.* 2010).

Mice with a mutation in *Npr3*, or *Npr3* knockout mice, all exhibit the same skeletal-overgrowth phenotype, resulting in an elongated body and thoracic kyphosis (Jaubert *et al.* 1999). Similarly, *Skm1* mice also exhibit these skeletal phenotypes. The *Skm1* mutation occurs within the membrane proximal region of the extracellular domain of *Npr3*, suggesting that ligand binding may be altered in these mice. A mutation in this domain would prevent clearance of the NPs and result in their accumulation. Transgenic mice that overexpress the brain NP or C-type NP ligand exhibit elongated bones and skeletal overgrowth (Suda *et al.* 1998; Yasoda *et al.* 2004). In addition, mice that overexpress osteocrin have elongated bones and exhibit a kyphosis phenotype (Moffatt *et al.* 2007). Together, these data suggest that an accumulation of NPR-C ligands in the *Skm1* mice may contribute to the skeletal phenotype. More recently, using a genome-wide analysis, *NPR3* was shown to be associated with overall body height and trunk length in humans (Soranzo *et al.* 2009). Whether the I384N mutation observed in the *Skm1* mice correlates to a SNP in human patients with kyphosis or in a subset of individuals with increased torso length remains to be determined.

The gene underlying the mutation in *Skm2* mice, *Flnb*, encodes filamin B, a member of a family of cytoplasmic proteins including filamin A and filamin C. These proteins provide a scaffold for the cytoskeletal network by crosslinking actin to the cytoskeleton, thereby regulating intracellular signaling and protein trafficking (Stossel *et al.* 2001). The *Skm2* mutation lies within a highly conserved hydrophobic region in the actin-binding site 2 (ABS2), which corresponds to the last α-helix of the calponin homology domain 1, and is critical for the binding of F-actin (van der Flier and Sonnenberg 2001). Mice that lack functional *Flnb* have been generated that exhibit impaired development of the microvascular and skeletal systems, including lack of invertebral discs and kyphotic and scoliotic spines (Farrington-Rock *et al.* 2008; Lu *et al.* 2007; Zhou *et al.* 2007). The mutation in *Skm2* (S96P) recapitulates the phenotype observed in the knockout mice, suggesting that this residue is critical for the function of FLNB. Interestingly, *Flnb* knockout is associated with embryonic lethality beginning at E11.5 and <3% of *Flnb^−/−^* survive to birth, with all mice being killed by 4 weeks of age because of impaired movement and reduced body weight (Zhou *et al.* 2007). Although *Skm2* mice are viable, the occurrence of the mutant phenotype (eight mutants in 84 pups) suggests that there may be some embryonic lethality in the *Skm2* mice as well.

Mutations in human *FLNB* have been found in various skeletal disorders with vertebral abnormalities, including spondylocarpotarsal synostosis, boomerang dysplasia, Larsen syndrome, and atelosteogenesis III. These disorders are characterized by vertebral fusions, abnormal spinal segmentation, and skeletogenesis (Krakow *et al.* 2004). Consistent with the *Flnb* knockout mice, the *Skm2* phenotype resembles that seen in human vertebral malformation syndromes involving this gene. To our knowledge, this is the first chemically induced allele of *Flnb* that has been reported.

Our findings emphasize the utility of ENU mutagenesis for the identification of genes important in spinal formation. Comparisons of mouse phenotypes with clinical descriptions will facilitate a better understanding of the genetic component of vertebral malformations.
